# The Story of the Middlesbrough Small-Pox Epidemic

**Published:** 1898-12-24

**Authors:** 


### THE STORY OF THE MIDDLESBROUGH SMALL-POX EPIDEMIC.

In the very interesting story of the Middlesbrough small-pox epidemic by Dr. Charles V. Dingle, medical officer of health of Middlesbrough, which appears in this month's *Public Health*, there is a table giving an analysis of the marks and quality of the vaccination of the 1,213 vaccinated persons who were attacked during the outbreak. Of the 1,213 vaccinated cases which were attacked, 183 had 1 mark (158 good and 25 bad), 454 had 2 marks (358 good and 96 bad), 231 had 3 marks (197 good and 34 bad), and 345 had 4 marks (279 good and 66 bad). Turning to another point, however, the story of the Middlesbrough epidemic is very instructive as to the immense importance of vaccination, not only in preventing attack, but in modifying the disease and robbing it of its fatality. At all ages the fatality per cent. of attacks was 8·8 among the vaccinated, and 47·4 among the unvaccinated. But that is not everything. If we accept it as a fact that the protective influence of vaccination gradually fades away, or, at any rate, greatly lessens with lapse of years, and therefore for the sake of keeping within a reasonable distance of the primary vaccination, take only the cases which occurred under 15 years of age, we find the following remarkable series of percentages of fatality among the vaccinated and the unvaccinated at the different ages: Under 1 year old, vaccinated, 0·0; unvaccinated, 71·4; from 1 to 5 years old, vaccinated, 0·0; unvaccinated, 44·4; from 5 to 10 years old, vaccinated, 0·0; unvaccinated, 43·5; and from 10 to 15 years old, vaccinated, 1·6; unvaccinated, 19·0. After that age the fatality of the disease in both classes seems to rise with increasing age; but within the first dozen years or so of vaccination no room is left for doubt as to the modifying power that the operation has upon such cases as are not entirely protected from the disease.

